# GPT-5–assisted versus expert surgeon refractive planning in smooth incision lenticule keratomileusis (SILK): comparative analysis and visual outcomes

**DOI:** 10.1007/s10792-026-04084-w

**Published:** 2026-05-02

**Authors:** Pier Luigi Surico, Tsung-Hsien Tsai, David Hui-Kang Ma, Chao Kai Chang, Gabriele Gallo Afflitto, Hung-Yu Lin, Chih-Chin Pan, Antonio Perri, Antonio Di Zazzo, Paolo Lanzetta, Chi-Chin Sun

**Affiliations:** 1 Department of Organs of Sense, Sapienza University, 00185 Rome, Italy; 2 Department of Medicine-Ophthalmology, University of Udine, Udine, Italy; 3 Department of Ophthalmology, Chang Gung Memorial Hospital, No. 222 Maijin Rd., Anle Dist., Keelung, 204 Taiwan; 4 Department of Ophthalmology, Chang Gung Memorial Hospital, Linkou, Taiwan; 5 Istituto Europeo di Microchirurgia Oculare-IEMO, Udine, Italy; 6 Nobel Eye Institute, Taipei, Taiwan; 7 Graduate Institute of Clinical Medicine, Kaohsiung Medical University, Kaohsiung, Taiwan; 8 Moorfields Eye Hospital NHS Foundation Trust, London, UK; 9 Department of Ophthalmology, Show Chwan Memorial Hospital, Changhua, Taiwan; 10 Department of Post-Baccalaureate Medicine, College of Medicine, National Chung Hsing University, Taichung, Taiwan; 11 Sun Ming Eye Clinic, Kaohsiung, Taiwan; 12 Department of Ophthalmology, Fondazione Policlinico Universitario Campus Bio-Medico, Rome, Italy; 13 School of Medicine, College of Medicine, Chang Gung University, Taoyuan, Taiwan

**Keywords:** Artificial intelligence, Large language models, GPT-5, Refractive surgery, Smooth incision lenticule keratomileusis, Lenticule extraction

## Abstract

**Purpose:**

To assess the performance of GPT-5 in refractive surgery planning by comparing its recommendations with expert surgeons and reporting visual outcomes of surgeon-planned Smooth Incision Lenticule Keratomileusis (SILK) procedures.

**Methods:**

This retrospective, observational study included 134 eyes from 67 patients who underwent SILK procedure using the ELITA femtosecond laser platform from January 2024 to September 2025. GPT-5 generated refractive plans based on manifest and cycloplegic refraction, autorefractometry, visual acuity, keratometry, and pachymetry. These were compared with surgeon-derived values using paired t-tests and correlation analysis. Preoperative and postoperative (1- and 3-month) uncorrected and corrected distance visual acuity (UDVA, CDVA), mean keratometry (Km), and thinnest corneal point were analyzed. Axis concordance was quantified as the absolute angular deviation (Δ Axis) between AI- and surgeon-planned astigmatism axes.

**Results:**

AI-generated spherical corrections were more conservative than surgeons’ (− 4.29 ± 2.42 D vs. − 4.70 ± 1.98 D; *p* = 0.0025), whereas cylindrical power did not differ significantly (p = 0.9241). The mean Δ Axis was 61.9° ± 50.1°, indicating substantial misalignment. UDVA improved from 0.74 ± 0.36 logMAR preoperatively to − 0.06 ± 0.07 logMAR at 3 months (*p* < 0.0001), and postoperative UDVA did not differ significantly from preoperative CDVA, confirming excellent visual recovery.

**Conclusions:**

GPT-5 generated refractive plans that were partially consistent with expert surgeon decisions but lacked the clinical precision required for clinical implementation. In contrast, surgeon-planned SILK procedures achieved excellent visual acuity, corneal stability, and refractive predictability, reinforcing the safety and efficacy of Kerato Lenticule Extraction (KLEx) in real-world clinical practice.

**Supplementary Information:**

The online version contains supplementary material available at 10.1007/s10792-026-04084-w.

## Introduction

The integration of artificial intelligence (AI) into clinical medicine has rapidly accelerated, with transformative effects across diagnostic, surgical, and therapeutic domains [1]. In ophthalmology, AI-driven tools are increasingly employed in imaging interpretation, surgical planning, and clinical decision support systems [2–5]. Among these, large language models (LLMs) such as ChatGPT have gained widespread popularity due to their ability to process complex inputs, generate clinically coherent text, and synthesize extensive medical knowledge bases [6]. While these capabilities hold promise for clinical assistance, the deployment of general-purpose AI tools in high-precision fields such as refractive surgery requires cautious validation [7]. For early-career surgeons in particular, AI-based recommendations must be rigorously benchmarked to ensure they reflect accurate, evidence-based, and patient-specific decision-making.

Concurrently, the global rise in myopia, reaching epidemic proportions in East Asia and increasingly affecting Western populations, has driven demand for durable, sustainable, spectacle-free solutions through refractive surgery [8, 9]. The landscape of refractive surgery continues to evolve with increasing adoption of keratorefractive lenticule extraction (KLEx) techniques [10]. These procedures, which eliminate the need for a corneal flap, preserve corneal biomechanics and reduce the incidence of dry eye and flap-related complications typically associated with laser in situ keratomileusis (LASIK) [11]. Among these, Smooth Incision Lenticule Keratomileusis (SILK), performed with the ELITA femtosecond laser platform (Johnson & Johnson Surgical Vision, Inc., Milpitas CA, USA) represents a next-generation advancement offering improved precision in lenticule creation, smoother incision profiles, low energy delivery and enhanced predictability of outcomes [12, 13].

Despite growing enthusiasm for AI-assisted planning and increasing clinical adoption of SILK, no prior studies have examined the intersection of these innovations. Specifically, it remains unclear whether AI-based treatment planning, such as that proposed by ChatGPT, can reliably replicate or enhance the refractive planning decisions of experienced surgeons in the context of lenticule extraction. Prior studies have shown that GPT-5 outperforms earlier models and ophthalmology residents in cataract and refractive surgery-related queries, particularly in complex prompts, but its potential role in refractive surgery planning is yet to be delineated [14]. Given the highly individualized nature of refractive surgery and its narrow safety margins, empirical validation is critical before such tools can be responsibly integrated into clinical workflows.

In this study, we aimed to: (1) compare refractive treatment plans generated by GPT-5 with those developed by experienced refractive surgeons in patients undergoing SILK; and (2) report the clinical outcomes achieved using surgeon-led treatment plans.

## Methods

### Study design

This study was designed as a retrospective, observational, noninterventional analysis of patients who underwent refractive surgery with the SILK procedure performed using the ELITA femtosecond laser platform. Clinical records and surgical planning data were reviewed for all consecutive patients treated between January 2024 and September 2025 at tertiary ophthalmology center Chang Gung Memorial Hospital (Taipei, Linkou and Keelung branches, Taiwan), Century Eye Clinic (Taichung, Taiwan), and Nobel Eye Clinic (Taipei, Taiwan). Our primary objective was to compare refractive treatment parameters (sphere, cylinder, and astigmatic axis) generated by an AI model with those selected by experienced refractive surgeons. Importantly, AI-generated treatment plans were created retrospectively for research comparison only and were not used to guide, modify, or influence any surgical procedures. All reported postoperative visual and refractive outcomes derive exclusively from surgeon-planned and surgeon-executed treatments.

### Patient selection and preoperative evaluation

Eligible patients were those who sought surgical correction for myopia or myopic astigmatism and underwent the SILK procedure during the study period. Inclusion criteria consisted of refractive stability for at least one year prior to surgery, no evidence of corneal ectatic disorders, and sufficient corneal thickness to allow safe lenticule extraction. Patients with a history of ocular surgery, clinically significant ocular surface disease, or systemic conditions known to impair corneal healing were excluded [15].

All patients underwent a comprehensive preoperative ophthalmic examination. This included measurement of uncorrected and corrected distance visual acuity (UDVA and CDVA), with visual acuity recorded in decimal notation and subsequently converted into logarithm of the minimum angle of resolution (logMAR) units and Snellen for analysis. The latter being a linear, continuous scale, it allowed for valid parametric statistical methods. Manifest and cycloplegic refraction were performed, with sphere, cylinder, and axis recorded. From these measurements, the spherical equivalent (SE) was calculated as the spherical power plus half the cylindrical power. Autorefractometer data were recorded. Corneal tomography was performed using a Scheimpflug-based system (Pentacam® HR, Oculus GmbH, Germany), from which keratometric parameters including flat keratometry (K1), steep keratometry (K2), maximum keratometry (Kmax), and mean keratometry (Km) were extracted. The orientation of the corneal astigmatism axis was also recorded. Pachymetric analysis using Scheimpflug-based system was carried out to identify the thinnest corneal point, which was used as the reference safety parameter, rather than central corneal thickness [16]. Slit-lamp biomicroscopy and dilated fundus examination were performed to exclude anterior and posterior segment pathology. As far as axis measurements are concerned, all values were recorded using a standardized 0°–180° ophthalmic notation format. Left and right eyes were analyzed using the same conventional axis notation without mirror transformation.

### Surgical planning and AI-assisted simulation

For each patient, two parallel treatment plans were generated. The first plan was prepared by the treating refractive surgeon, who evaluated clinical data to select the correction values. The second plan was generated using GPT-5 (OpenAI, 2025), a state-of-the-art large language model capable of multimodal reasoning. GPT-5 was prompted with a structured dataset that included manifest refraction, cycloplegic refraction, autorefractometer measurements, keratometry values (K1, K2, Km), astigmatism axis, corneal thinnest point thickness, and baseline UDVA and CDVA. The model was instructed to simulate the reasoning process of an experienced refractive surgeon, with particular attention to accuracy of spherical and cylindrical correction and axis alignment. The GPT-5 output provided suggested values for sphere, cylinder, and axis of correction for each patient, which were then documented for comparative analysis against the surgeon’s plan. Importantly, the AI-generated plan was produced retrospectively from the same preoperative data and was not used during the actual surgery.

### Surgical technique

All procedures were performed with the ELITA femtosecond laser platform, following the standardized SILK technique [17]. This flapless procedure involves the creation and removal of an intrastromal refractive lenticule through a small incision, typically located in the superior cornea, thereby preserving anterior stromal architecture and maintaining corneal biomechanical stability. The surgical parameters, including optical zone, cap thickness, and lenticule geometry, were selected according to the surgeon’s preoperative plan. AI-generated parameters were not used intraoperatively. All interventions were performed under topical anesthesia and were uneventful. Postoperative management included a short course of topical antibiotics and corticosteroids tapered over two weeks, followed by lubricants as required.

### Postoperative follow-up

Patients were examined at one and three months postoperatively. At each visit, UDVA and CDVA were assessed and recorded. Corneal tomography with Pentacam® was performed to monitor keratometric parameters, astigmatism axis, corneal thinnest point thickness, and astigmatism axis alignment. All postoperative visual and refractive outcomes reported in this study reflect the results of surgeon-planned procedures only.

### Analysis of axis concordance

A central focus of the analysis was the comparison between AI-predicted and surgeon-planned astigmatic axis. To quantify the degree of concordance, the absolute angular difference (Δ Axis) was calculated. This value was defined as the smallest angular separation between the two axes on a 0°–180° scale, expressed mathematically as: Δ Axis = min(|Axis_1_ − Axis_2_|, 180° −|Axis_1_ − Axis_2_|) [18, 19]. This method ensures that the circular nature of astigmatic axis measurement is properly accounted for and prevents overestimation of angular discrepancies.

### Statistical analysis

All statistical analyses were performed using GraphPad Prism (version 10.1, GraphPad Software, San Diego, CA) and Microsoft Excel (Microsoft 365, v16.x; Microsoft Corp., Redmond, WA). Continuous variables were expressed as mean values with standard deviation (SD). Normality of data distribution was assessed using the Shapiro–Wilk test. Paired t-tests were applied to compare AI-assisted versus surgeon-planned parameters for sphere, cylinder, and SE. Repeated measures analysis of variance (ANOVA) was used to evaluate changes in visual acuity, spherical equivalent, keratometry, and corneal thickness across the three time points (preoperative, one month, and three months). For axis analysis, the mean Δ Axis and its distribution within clinically relevant thresholds (≤ 5°, ≤ 10°, and ≤ 15°) were reported. A p value < 0.05 was considered statistically significant.

## Results

### Patient demographics and baseline characteristics

A total of 134 eyes from 67 patients were included in this retrospective analysis of AI-assisted ELITA-SILK refractive procedures. Among these, 36 patients (53.7%) were female and 31 (46.3%) were male. The mean age at the time of surgery was 32.4 ± 6.7 years (range: 21–47 years). Mean preoperative manifest spherical equivalent (SE) refractive error was –4.21 ± 1.87 D, with a manifest sphere of − 3.81 ± 1.79 D and a cylinder of − 0.80 ± 0.69 D. Corresponding cycloplegic SE was − 4.09 ± 1.82 D, suggesting minimal accommodative change. Baseline UDVA was 0.74 ± 0.36 logMAR, while CDVA was − 0.07 ± 0.06 logMAR, indicating a high baseline corrected visual acuity. Preoperative corneal topography, showed a mean K1 of 43.23 ± 1.23 D, K2 of 44.57 ± 1.24 D, and a mean Km of 43.05 ± 1.16 D. The mean corneal pachymetry, recorded at the thinnest point, was 548.9 ± 32.6 µm (Table 1).

**Table 1:** Baseline demographic and clinical characteristics of the patients

Number of patients	67
Number of eyes	134
Female patients, n (%)	36 (53.7%)
Male patients, n (%)	31 (46.3%)
Age at surgery (years)	32.4 ± 6.7 (range: 21–47)
Preoperative manifest SE (D)	− 4.21 ± 1.87
Preoperative manifest sphere (D)	− 3.81 ± 1.79
Preoperative manifest cylinder (D)	− 0.80 ± 0.69
Cycloplegic SE (D)	− 4.09 ± 1.82
UDVA (logMAR)	0.74 ± 0.36
CDVA (logMAR)	− 0.07 ± 0.06
Flat K (K1, D)	43.21 ± 1.23
Steep K (K2, D)	44.57 ± 1.24
Mean keratometry (Km, D)	43.05 ± 1.16
Thinnest corneal pachymetry (μm)	548.9 ± 32.6

SE = Spherical equivalent; UDVA = Uncorrected distance visual acuity; CDVA = Corrected distance visual acuity; K1 = Flat keratometry; K2 = Steep keratometry; Km = Mean keratometry; D = Diopters; logMAR = Logarithm of the minimum angle of resolution. All data are presented as mean ± standard deviation unless otherwise indicated

### Comparison of AI and surgeon treatment planning

**Fig. 1:**
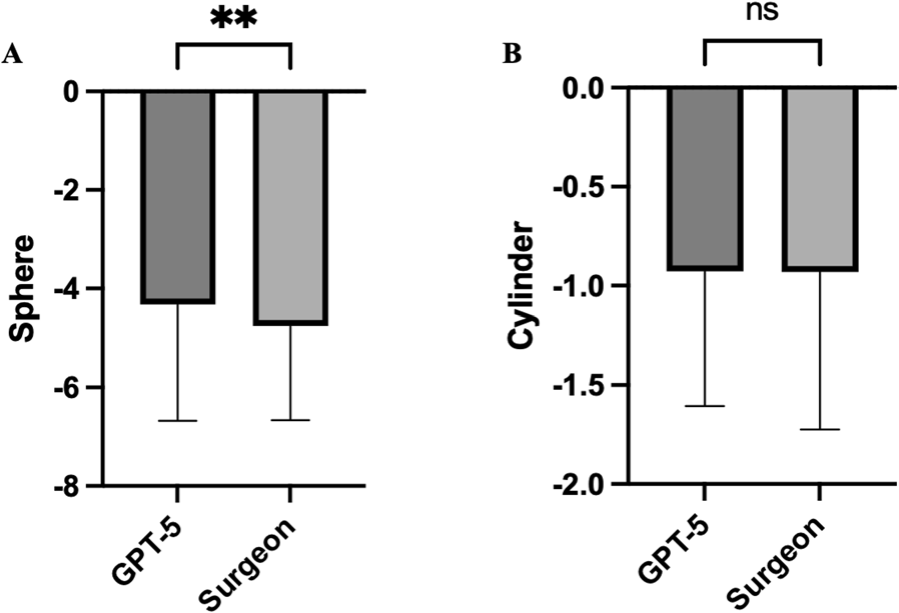
Comparison of GPT-5–assisted and surgeon-planned refractive corrections. **A** Planned spherical correction (D). GPT-5: − 4.29 ± 2.42 vs. Surgeons: − 4.70 ± 1.98; the spherical component of the AI-assisted plans was significantly less myopic compared to surgeon-derived corrections (mean difference: − 0.41 D, p = 0.0025, paired t-test; r = 0.77). **B** Planned cylindrical correction (D). GPT-5: − 0.91 ± 0.69 vs Surgeons: − 0.92 ± 0.80. No statistically significant difference was observed in the cylindrical component (*p* = 0.9241), with a strong correlation (r = 0.92) between the two methods. Values are mean ± SD; error bars represent SD

A total of 134 eyes were included in the comparative analysis of refractive planning using GPT-5 versus experienced refractive surgeons. The spherical component of the planned correction was found to be significantly different between the two approaches. The AI-assisted plans (mean sphere: − 4.29 ± 2.42 D) were slightly more conservative than those of the surgeons (mean sphere: − 4.70 ± 1.98 D). This difference reached statistical significance (*p* = 0.0025, paired t-test), with a mean bias of − 0.41 D in favor of the GPT-5 suggestions. The correlation between paired values was strong (r = 0.77), indicating consistent intra-subject matching (Fig. 1A).

In contrast, the cylindrical component of the planned correction showed no statistically significant difference between AI and surgeon plans. The mean cylindrical correction proposed by GPT-5 was − 0.91 ± 0.69 D compared to − 0.92 ± 0.80 D by surgeons (*p* = 0.9241, paired t-test). The negligible mean difference and high correlation confirmed that both approaches yielded nearly identical astigmatism magnitudes (Fig. 1B).

The angular difference between AI-planned and surgeon-planned astigmatism axis was analyzed using absolute angular deviation (Δ Axis). The mean Δ Axis was 61.9° ± 50.1°. Only 9.0% of eyes had a Δ Axis within 5°, 12.8% within 10°, and 18.0% within 15°, indicating low concordance in axis orientation. While the cylindrical magnitudes were highly aligned, the AI frequently suggested different axis orientations than the human planner, potentially reflecting a distinct prioritization of symmetry or topographic features in the AI algorithm.

### Visual outcomes

**Fig. 2:**
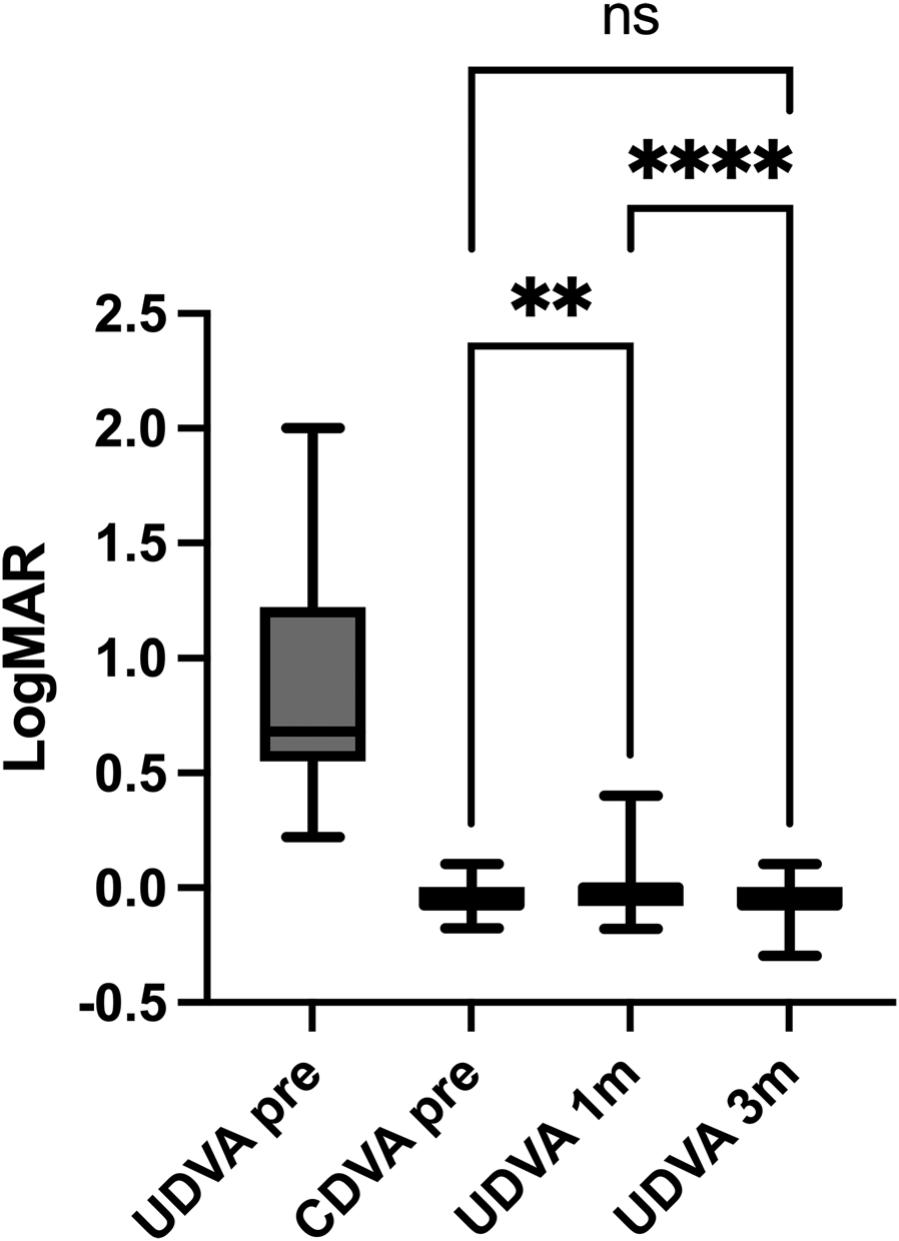
Visual acuity outcomes following ELITA-SILK. Uncorrected distance visual acuity (UDVA) improved postoperatively at both 1 and 3 months (*p* < 0.0001), showing no statistically significant difference from preoperative corrected distance visual acuity (CDVA), thus confirming refractive efficacy and safety. UDVA improved to − 0.03 ± 0.08 logMAR at 1 month and − 0.06 ± 0.07 at 3 months (repeated-measures ANOVA *p* < 0.0001; Tukey post-hoc *p* < 0.0001 vs. preoperative for both). Preoperative CDVA was − 0.07 ± 0.06 logMAR and remained stable postoperatively. Postoperative UDVA did not differ significantly from preoperative CDVA. Values are mean ± SD

**Fig. 3:**
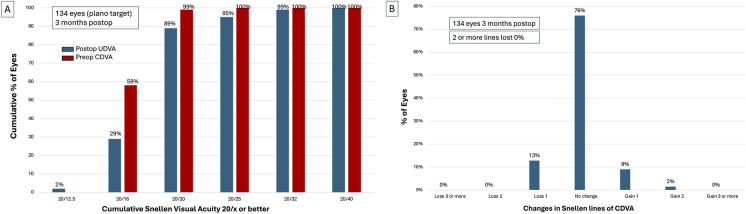
A. Cumulative proportion of eyes achieving 20/x or better for uncorrected distance visual acuity (UDVA) at 3 months vs. pre-op corrected distance visual acuity (CDVA) in Snellen chart: 89% achieved 20/20 or better; 99% achieved 20/32 or better. Postoperative UDVA approximated preoperative CDVA. B. CDVA Snellen lines changes from pre-op to 3 months post-op: 0% lost ≥ 2 lines; 13% lost 1 line; 76% unchanged; 9% gained 1 line; 2% gained 2 lines

UDVA improved significantly following the surgeon-assisted ELITA-SILK procedure to − 0.03 ± 0.08 logMAR at 1 month and further to − 0.06 ± 0.07 logMAR at 3 months postoperatively. A repeated-measures one-way ANOVA demonstrated a significant difference across time points (*p* < 0.0001), and post-hoc Tukey tests confirmed a significant improvement from preoperative UDVA to both 1-month and 3-month postoperative measurements (*p* < 0.0001 for both comparisons). CDVA remained stable throughout the observation period. The mean preoperative CDVA was − 0.07 ± 0.06 logMAR, with no statistically significant difference when compared to postoperative CDVA at 1 or 3 months (Table 2). Importantly, postoperative UDVA was not significantly different from preoperative CDVA, underscoring the efficacy of the procedure in restoring visual function without the need for correction (Fig. 2). At 3 months, cumulative Snellen curves confirm UDVA closely approaching pre-op CDVA (Fig. 3A) and the safety histogram indicates minimal loss of lines (Fig. 3B).

**Table 2:** Postoperative clinical outcomes

Parameter	Baseline	1 Month	3 Months	*p*-Value
UDVA (logMAR)	0.74 ± 0.36	− 0.03 ± 0.08	− 0.06 ± 0.07	< 0.0001
CDVA (logMAR)	− 0.07 ± 0.06	− 0.04 ± 0.07	− 0.07 ± 0.08	0.999
Thinnest point corneal thickness (µm)	548.9 ± 32.6	449.4 ± 34.1	448.6 ± 34.7	< 0.0001
Km (D)	43.05 ± 1.16	39.40 ± 1.35	39.30 ± 1.35	< 0.0001

UDVA: Uncorrected distance visual acuity; CDVA: Corrected distance visual acuity; LogMAR: Logarithm of the minimum angle of resolution; Km: Mean keratometry; D: Diopters

### Anatomical parameters

Mean thinnest point corneal thickness significantly decreased after surgery. Preoperative measurements averaged 548.9 ± 32.6 µm, reducing to 449.4 ± 34.1 µm at 1 month and 448.6 ± 34.7 µm at 3 months postoperatively. The reductions from baseline to both postoperative time points were statistically significant (*p* < 0.0001), while no significant change was observed between the 1- and 3-month measurements (*p* = 0.4016), indicating early structural stabilization of the cornea (Table 2).

Keratometric flattening was also observed following treatment. Km decreased from a preoperative mean value of 43.05 ± 1.16 D to 39.40 ± 1.35 D at 1 month and 39.30 ± 1.35 D at 3 months. The differences from baseline to each postoperative time point were statistically significant (*p* < 0.0001), whereas no further significant flattening occurred between the two postoperative time points (*p* = 0.7597), suggesting stable refractive changes (Table 2).

## Discussion

The increasing availability of high-resolution imaging data and quantitative biometric parameters makes the ophthalmology field particularly well-suited for AI-driven applications [19, 20]. In the context of refractive surgery, where precise interpretation of corneal tomography, pachymetry, refractive error, and biomechanical factors is essential, AI-assisted models have been shown to support surgical planning [21]. However, to our knowledge, no prior study has evaluated the integration of a GPT-5-powered AI planning system against experienced refractive surgeons in femtosecond laser-assisted lenticule extraction using the ELITA-SILK platform. Indeed, literature has focused on other KLEx techniques such as SMILE on decision support [22]. Our results were multifaceted. First, the clinical outcomes of surgeon-driven procedure, evaluated by visual acuity, corneal curvature, and structural thickness, confirmed the safety, efficacy, and predictability of the SILK approach for refractive correction. UDVA improved significantly from 0.74 logMAR preoperatively to − 0.03 and − 0.06 logMAR at 1 and 3 months (*p* < 0.0001), reaching or surpassing preoperative CDVA in most cases. CDVA remained stable postoperatively, with no significant losses, confirming the efficacy and safety of the technique. [23]. Second, in terms of anatomical changes, keratometric flattening was observed, with mean Km decreasing from 43.05 D to 39.30 D at 3 months, as expected. Similarly, corneal thinning was consistent with tissue removal and stabilized early after the procedure. These findings reinforce the predictability and structural advantages of KLEx over flap-based procedures, including faster biomechanical recovery and lower risk of dry eye, particularly in eyes with thinner corneas or high refractive error [10, 11, 13, 23, 24]. The integration of GPT-5 in this context represents a proof-of-concept for the use of LLMs to support real-world, high-precision surgical planning [24]. Third, GPT-5 plans showed strong correlation with surgeons for cylindrical power (r = 0.92) but exhibited systematic differences in spherical power (mean Δ =  − 0.41 D, *p* = 0.0025), with AI tending toward more conservative corrections. AI-based nomogram studies in SMILE demonstrated improved refractive accuracy and reduced spherical undercorrection compared with surgeon-set planning. For instance, Cui et al. reported a mean spherical equivalent error of − 0.09 D (*p* < 0.001) using a machine learning nomogram versus − 0.23 D (*p* < 0.001) with surgeon-set planning [22]. However, their model was trained on structured SMILE outcome data, whereas our GPT-5 system is a general-purpose LLM without SILK-specific outcome calibration. Given that LLM performance depends on domain-specific training data, the absence of dedicated refractive datasets may partly explain the conservative spherical bias observed in our AI-generated plans. More notably, agreement in astigmatic axis orientation was limited (mean Δ axis = 61.9° ± 50.1), with fewer than 20% of cases within 15° of surgeon alignment, thus indicating that while GPT-5 can replicate general refractive trends, it lacks precision in angular and patient-specific decision-making. Refractive surgery remains a highly personalized and precision-driven field, in which even minimal (sub-diopter and sub-degree) variations can significantly influence postoperative outcomes. The discrepancies observed in AI-generated plans emphasize that current general LLMs cannot yet replace expert refractive surgeons in individualized planning. Dedicated ophthalmic training datasets, integration of biomechanical modeling, and validation in prospective clinical trials are prerequisites for safe AI deployment. To the best of our knowledge, there isn’t any prior retrospective, observational and multicentric study comparing an AI model vs Surgeon planning in the context of SILK procedure. Nevertheless, several limitations should be acknowledged. First, retrospective and multicenter design of the study may have introduced selection and measurement biases. Second, multiple surgeons across participating centers were involved in surgical planning. Although standardized institutional planning principles were followed, individual clinical judgment was applied. Potential inter-surgeon variability was acknowledged as reflective of real-world clinical practice. Third, GPT-5 was not specifically trained on ophthalmic datasets, and its integration was manual rather than embedded into a clinical decision-making interface. Fourth, long-term stability beyond 3 months and patient-reported outcomes were not assessed in this initial investigation. Fifth, the relatively short follow-up f the study limits the assessment of long-term outcomes, including refractive stability, regression, and enhancement rates. Future studies with extended follow-up are needed to better characterize the durability of SILK procedures. In parallel, the development of domain-specific AI systems trained on curated refractive datasets may provide valuable support for surgical planning, reduce variability, and offer second-opinion guidance, particularly in complex cases or for less experienced surgeons. However, rigorous validation and appropriate ethical oversight will be essential prior to clinical implementation. Overall, these findings should be considered a preliminary proof-of-concept, highlighting both the potential and current limitations of general-purpose AI models in ophthalmology.

## Conclusions

In this study, we showed that GPT-5–assisted refractive planning yielded refractive targets comparable to those of experienced surgeons in terms of cylindrical correction; however, significant discrepancies remained in spherical power and axis alignment. These findings highlight the limitations of general-purpose LLMs in highly customized surgical planning. Conversely, surgeon-planned lenticule extraction using the ELITA-SILK platform resulted in excellent visual acuity, early structural stability, and refractive predictability, reinforcing the clinical efficacy and safety of KLEx in real-world refractive surgery. While LLMs offer promising support potential, their integration into refractive surgery demands purpose-built training and rigorous validation to meet the precision standards of this field.

## Supplementary Information

Below is the link to the electronic supplementary material.

Supplementary file1 (DOCX 195 KB)

## Data Availability

The dataset generated during the current study is available from the corresponding author on reasonable request.
